# (*E*)-2,4-Dimethyl-*N*′-(2-methyl­benzyl­idene)benzohydrazide

**DOI:** 10.1107/S1600536813004388

**Published:** 2013-02-20

**Authors:** Muhammad Taha, Nor Hadiani Ismail, Faridahanim Mohd Jaafar, Khalid M. Khan, Sammer Yousuf

**Affiliations:** a Atta-ur-Rahman Institute for Natural Product Discovery, Universiti Teknologi MARA (UiTM), Puncak Alam Campus, 42300 Bandar Puncak Alam, Selangor, Malaysia; b Faculty of Applied Science, Universiti Teknologi MARA (UiTM), 40450 Shah Alam, Malaysia; c H.E.J. Research Institute of Chemistry, International Center for Chemical and Biological Sciences, University of Karachi, Karachi 75270, Pakistan

## Abstract

In the title benzoyl­hydrazide derivative, C_17_H_18_N_2_O, the dihedral angle between the benzene rings is 88.45 (8)° and the azomethine double bond adopts an *E* conformation. In the crystal, mol­ecules are linked by N—H⋯O and C—H⋯O hydrogen bonds, forming a chain along the *b* axis.

## Related literature

For the applications and biological activity of Schiff bases, see: Musharraf *et al.* (2012 ▶); Khan *et al.* (2012 ▶). For the crystal structures of related compounds, see: Taha *et al.* (2012*a* ▶,*b* ▶); Naz *et al.* (2012 ▶).
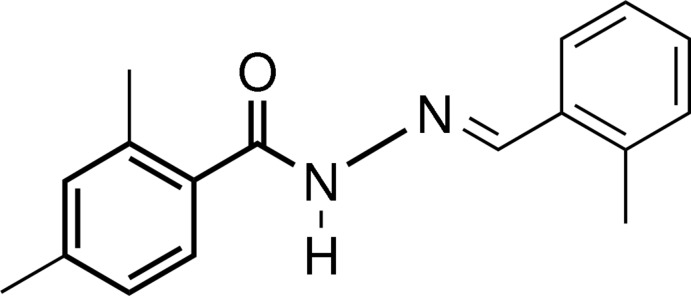


## Experimental

### 

#### Crystal data

C_17_H_18_N_2_O
*M* *_r_* = 266.33
Orthorhombic, 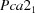

*a* = 26.1151 (10) Å
*b* = 4.9484 (2) Å
*c* = 11.3933 (4) Å
*V* = 1472.33 (10) Å^3^
*Z* = 4
Mo *K*α radiation
μ = 0.08 mm^−1^
*T* = 273 K
0.56 × 0.55 × 0.23 mm

#### Data collection

Bruker SMART APEX CCD area-detector diffractometer
Absorption correction: multi-scan (*SADABS*; Bruker, 2000 ▶) *T*_min_ = 0.959, *T*_max_ = 0.983
8023 measured reflections
2577 independent reflections
2483 reflections with *I* > 2σ(*I*)
*R*_int_ = 0.021

#### Refinement

*R*[*F* ^2^ > 2σ(*F* ^2^)] = 0.030
*wR*(*F* ^2^) = 0.080
*S* = 1.04
2577 reflections
189 parameters
1 restraint
H atoms treated by a mixture of independent and constrained refinement
Δρ_max_ = 0.12 e Å^−3^
Δρ_min_ = −0.14 e Å^−3^

### 

Data collection: *SMART* (Bruker, 2000 ▶); cell refinement: *SAINT* (Bruker, 2000 ▶); data reduction: *SAINT*; program(s) used to solve structure: *SHELXS97* (Sheldrick, 2008 ▶); program(s) used to refine structure: *SHELXL97* (Sheldrick, 2008 ▶); molecular graphics: *SHELXTL* (Sheldrick, 2008 ▶); software used to prepare material for publication: *SHELXTL*, *PARST* (Nardelli, 1995 ▶) and *PLATON* (Spek, 2009 ▶).

## Supplementary Material

Crystal structure: contains datablock(s) global, I. DOI: 10.1107/S1600536813004388/is5246sup1.cif

Structure factors: contains datablock(s) I. DOI: 10.1107/S1600536813004388/is5246Isup2.hkl

Supplementary material file. DOI: 10.1107/S1600536813004388/is5246Isup3.cml

Additional supplementary materials:  crystallographic information; 3D view; checkCIF report

## Figures and Tables

**Table 1:** Hydrogen-bond geometry (Å, °)

*D*—H⋯*A*	*D*—H	H⋯*A*	*D*⋯*A*	*D*—H⋯*A*
N1—H1*A*⋯O1^i^	0.833 (15)	2.000 (15)	2.8150 (14)	166.1 (14)
C8—H8*A*⋯O1^i^	0.93	2.52	3.2696 (19)	138

Symmetry code: (i) 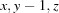.

**Fig. 1.**
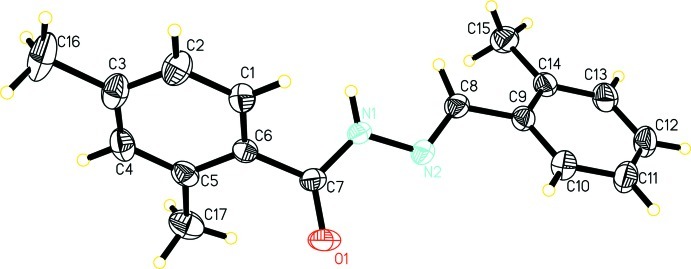
The molecular structure of the title compound, with displacement ellipsoids drawn at 30% probability level.

**Fig. 2.**
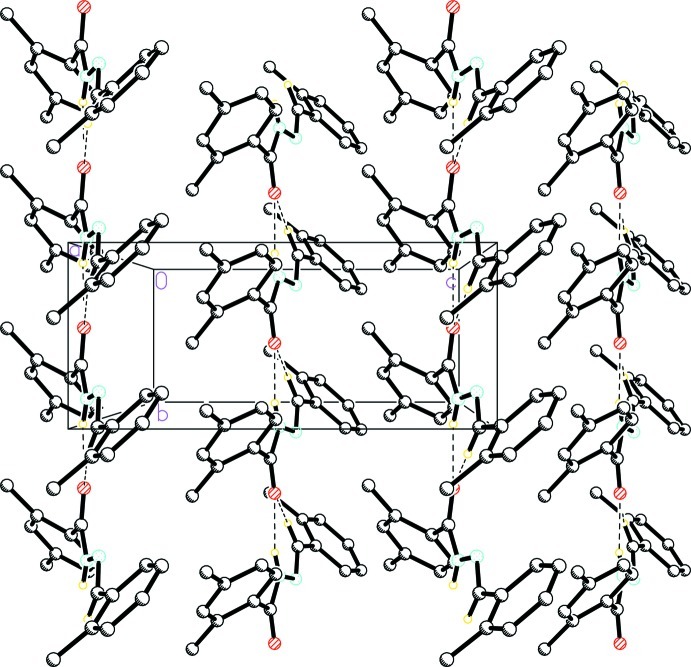
A crystal packing diagram of the title compound. Only hydrogen atoms involved in the hydrogen bonds (dashed lines) are shown.

## supplementary crystallographic information

### Comment

Benzohydrazides represents an important class of organic compounds with a wide range of biological applications (Musharraf *et al.*, 2012; Khan *et al.*, 2012). The title compound was obtained in continuation of our work to synthesize and study the biological activities of benzohydrazide derivatives. Previously, we have published crystal structures of many benzohydrazides derivatives with different substitution pattern on two phenyl rings (Taha *et al.*, 2012*a*,*b*; Naz *et al.*, 2012). In the title compound two methyl substituted phenyl rings (C1–C6 and C9–C14) are each planner with dihedral angle of 88.45 (8)° between them. The bond lengths and angles were found to be similar as in structurally related benzohydrazide derivatives (Taha *et al.*, 2012*a*,*b*; Naz *et al.*, 2012). The crystal structure stabilize by intermolecular N1—H1A···O1^i^ and C8—H8A···O1^i^ interactions to form a chain along the *b* axis (symmetry code as in Table 1).

### Experimental

The title compound was synthesized by using (0.328 g) 2 mmol of 2,4-dimethylbenzohydrazide and (0.240 g) 2 mmol 2-methylbenzaldehyde as starting material under same conditions and solvents as described in our previous publications (Taha *et al.*, 2012*a*,*b*; Naz *et al.*, 2012). The compound was recrystallized by dissolving in methanol to obtain colorless needles (0.474 g, 89% yield). All chemicals were purchased by sigma Aldrich Germany.

### Refinement

H atoms on methyl and phenyl groups were positioned geometrically with C—H = 0.96 and 0.93 Å, respectively, and constrained to ride on their parent atoms with *U*_iso_(H) = 1.5*U*_eq_(C_methyl_) or 1.2*U*_eq_(C_phenyl_). A rotating group model was applied to the methyl groups. The H atoms on the nitrogen was located in a difference Fourier map and refined isotropically [N—H = 0.832 (15) Å]. The Hooft y parameter was -0.3 (5).

### Crystal data

**Table:**

C_17_H_18_N_2_O	*F*(000) = 568
*M**_r_* = 266.33	*D*_x_ = 1.202 Mg m^−^^3^
Orthorhombic, *P**c**a*2_1_	Mo *K*α radiation, λ = 0.71073 Å
Hall symbol: P 2c -2ac	Cell parameters from 4590 reflections
*a* = 26.1151 (10) Å	θ = 2.4–27.4°
*b* = 4.9484 (2) Å	µ = 0.08 mm^−^^1^
*c* = 11.3933 (4) Å	*T* = 273 K
*V* = 1472.33 (10) Å^3^	Block, colorles
*Z* = 4	0.56 × 0.55 × 0.23 mm

### Data collection

**Table:**

Bruker SMART APEX CCD area-detector diffractometer	2577 independent reflections
Radiation source: fine-focus sealed tube	2483 reflections with *I* > 2σ(*I*)
Graphite monochromator	*R*_int_ = 0.021
ω scan	θ_max_ = 25.5°, θ_min_ = 2.4°
Absorption correction: multi-scan (*SADABS*; Bruker, 2000)	*h* = −25→31
*T*_min_ = 0.959, *T*_max_ = 0.983	*k* = −5→5
8023 measured reflections	*l* = −13→13

### Refinement

**Table:**

Refinement on *F*^2^	Secondary atom site location: difference Fourier map
Least-squares matrix: full	Hydrogen site location: inferred from neighbouring sites
*R*[*F*^2^ > 2σ(*F*^2^)] = 0.030	H atoms treated by a mixture of independent and constrained refinement
*wR*(*F*^2^) = 0.080	*w* = 1/[σ^2^(*F*_o_^2^) + (0.0459*P*)^2^ + 0.1205*P*] where *P* = (*F*_o_^2^ + 2*F*_c_^2^)/3
*S* = 1.04	(Δ/σ)_max_ < 0.001
2577 reflections	Δρ_max_ = 0.12 e Å^−^^3^
189 parameters	Δρ_min_ = −0.14 e Å^−^^3^
1 restraint	Extinction correction: *SHELXTL* (Sheldrick, 2008), Fc^*^=kFc[1+0.001xFc^2^λ^3^/sin(2θ)]^-1/4^
Primary atom site location: structure-invariant direct methods	Extinction coefficient: 0.016 (2)

### Special details

**Table:**

Geometry. All e.s.d.'s (except the e.s.d. in the dihedral angle between two l.s. planes) are estimated using the full covariance matrix. The cell e.s.d.'s are taken into account individually in the estimation of e.s.d.'s in distances, angles and torsion angles; correlations between e.s.d.'s in cell parameters are only used when they are defined by crystal symmetry. An approximate (isotropic) treatment of cell e.s.d.'s is used for estimating e.s.d.'s involving l.s. planes.
Refinement. Refinement of *F*^2^ against ALL reflections. The weighted *R*-factor *wR* and goodness of fit *S* are based on *F*^2^, conventional *R*-factors *R* are based on *F*, with *F* set to zero for negative *F*^2^. The threshold expression of *F*^2^ > σ(*F*^2^) is used only for calculating *R*-factors(gt) *etc*. and is not relevant to the choice of reflections for refinement. *R*-factors based on *F*^2^ are statistically about twice as large as those based on *F*, and *R*- factors based on ALL data will be even larger.

### Fractional atomic coordinates and isotropic or equivalent isotropic displacement parameters (Å^2^)

**Table:**

	*x*	*y*	*z*	*U*_iso_*/*U*_eq_	
O1	0.40241 (5)	0.5493 (2)	0.43046 (18)	0.0938 (6)	
N1	0.38352 (4)	0.1086 (2)	0.44326 (11)	0.0438 (3)	
H1A	0.3925 (5)	−0.050 (3)	0.4301 (13)	0.039 (4)*	
N2	0.33498 (4)	0.1558 (2)	0.48752 (10)	0.0448 (3)	
C1	0.49419 (6)	0.0399 (3)	0.44104 (14)	0.0500 (3)	
H1B	0.4797	−0.0346	0.5082	0.060*	
C2	0.54235 (6)	−0.0412 (3)	0.40641 (15)	0.0600 (4)	
H2B	0.5601	−0.1679	0.4510	0.072*	
C3	0.56461 (6)	0.0633 (4)	0.30653 (15)	0.0605 (4)	
C4	0.53740 (6)	0.2556 (4)	0.24413 (14)	0.0588 (4)	
H4A	0.5524	0.3297	0.1775	0.071*	
C5	0.48869 (6)	0.3437 (3)	0.27612 (13)	0.0488 (4)	
C6	0.46699 (5)	0.2308 (3)	0.37737 (12)	0.0426 (3)	
C7	0.41514 (5)	0.3145 (3)	0.41905 (14)	0.0480 (3)	
C8	0.30625 (5)	−0.0517 (3)	0.49163 (13)	0.0459 (3)	
H8A	0.3181	−0.2157	0.4624	0.055*	
C9	0.25464 (5)	−0.0345 (3)	0.54203 (13)	0.0458 (3)	
C10	0.24424 (6)	0.1476 (3)	0.63203 (14)	0.0553 (4)	
H10A	0.2703	0.2579	0.6602	0.066*	
C11	0.19599 (7)	0.1674 (4)	0.68005 (15)	0.0656 (5)	
H11A	0.1893	0.2922	0.7392	0.079*	
C12	0.15778 (7)	0.0008 (4)	0.63967 (17)	0.0665 (5)	
H12A	0.1252	0.0108	0.6722	0.080*	
C13	0.16772 (6)	−0.1808 (3)	0.55115 (18)	0.0628 (4)	
H13A	0.1414	−0.2922	0.5248	0.075*	
C14	0.21564 (5)	−0.2029 (3)	0.50006 (14)	0.0500 (3)	
C15	0.22448 (7)	−0.3941 (3)	0.39994 (17)	0.0652 (4)	
H15A	0.1940	−0.4979	0.3862	0.098*	
H15B	0.2522	−0.5136	0.4189	0.098*	
H15C	0.2329	−0.2933	0.3306	0.098*	
C16	0.61613 (7)	−0.0348 (6)	0.2648 (2)	0.0969 (8)	
H16A	0.6383	−0.0606	0.3311	0.145*	
H16B	0.6308	0.0969	0.2129	0.145*	
H16C	0.6120	−0.2030	0.2239	0.145*	
C17	0.46107 (8)	0.5471 (4)	0.20175 (18)	0.0736 (5)	
H17A	0.4250	0.5091	0.2028	0.110*	
H17B	0.4735	0.5370	0.1226	0.110*	
H17C	0.4670	0.7251	0.2323	0.110*	

### Atomic displacement parameters (Å^2^)

**Table:**

	*U*^11^	*U*^22^	*U*^33^	*U*^12^	*U*^13^	*U*^23^
O1	0.0731 (8)	0.0328 (5)	0.1753 (17)	0.0011 (5)	0.0407 (10)	−0.0067 (8)
N1	0.0394 (6)	0.0335 (5)	0.0587 (7)	0.0032 (4)	0.0055 (5)	−0.0042 (5)
N2	0.0388 (6)	0.0451 (6)	0.0505 (6)	0.0038 (5)	0.0034 (5)	−0.0020 (5)
C1	0.0471 (8)	0.0556 (8)	0.0472 (8)	−0.0015 (6)	0.0033 (7)	0.0003 (7)
C2	0.0467 (8)	0.0745 (10)	0.0586 (10)	0.0108 (7)	−0.0058 (8)	−0.0036 (8)
C3	0.0421 (8)	0.0810 (11)	0.0583 (9)	−0.0034 (7)	0.0019 (8)	−0.0175 (8)
C4	0.0589 (10)	0.0728 (10)	0.0448 (8)	−0.0218 (8)	0.0111 (7)	−0.0100 (7)
C5	0.0555 (9)	0.0431 (7)	0.0477 (8)	−0.0127 (6)	−0.0011 (7)	−0.0043 (6)
C6	0.0429 (7)	0.0367 (6)	0.0482 (8)	−0.0057 (5)	0.0015 (6)	−0.0066 (5)
C7	0.0460 (8)	0.0349 (6)	0.0630 (9)	−0.0006 (5)	0.0038 (7)	−0.0038 (6)
C8	0.0415 (7)	0.0427 (6)	0.0534 (8)	0.0024 (6)	0.0011 (6)	−0.0018 (6)
C9	0.0414 (7)	0.0469 (7)	0.0492 (7)	0.0032 (6)	0.0032 (6)	0.0059 (6)
C10	0.0508 (9)	0.0632 (9)	0.0520 (8)	−0.0002 (7)	0.0027 (7)	−0.0012 (7)
C11	0.0663 (11)	0.0764 (11)	0.0541 (9)	0.0055 (9)	0.0177 (8)	−0.0022 (8)
C12	0.0499 (9)	0.0789 (11)	0.0707 (11)	0.0030 (8)	0.0207 (9)	0.0056 (9)
C13	0.0458 (8)	0.0650 (9)	0.0774 (11)	−0.0079 (7)	0.0060 (9)	0.0036 (9)
C14	0.0454 (8)	0.0459 (7)	0.0586 (8)	−0.0005 (6)	0.0026 (7)	0.0066 (6)
C15	0.0578 (9)	0.0578 (9)	0.0800 (12)	−0.0053 (7)	0.0007 (9)	−0.0111 (9)
C16	0.0501 (11)	0.150 (2)	0.0910 (16)	0.0072 (12)	0.0129 (10)	−0.0378 (15)
C17	0.0953 (15)	0.0592 (10)	0.0664 (11)	−0.0078 (9)	−0.0073 (11)	0.0132 (8)

### Geometric parameters (Å, º)

**Table:**

O1—C7	1.2156 (17)	C9—C14	1.400 (2)
N1—C7	1.3401 (17)	C10—C11	1.377 (2)
N1—N2	1.3842 (16)	C10—H10A	0.9300
N1—H1A	0.832 (15)	C11—C12	1.374 (3)
N2—C8	1.2723 (18)	C11—H11A	0.9300
C1—C2	1.378 (2)	C12—C13	1.376 (3)
C1—C6	1.387 (2)	C12—H12A	0.9300
C1—H1B	0.9300	C13—C14	1.384 (2)
C2—C3	1.378 (3)	C13—H13A	0.9300
C2—H2B	0.9300	C14—C15	1.500 (2)
C3—C4	1.384 (3)	C15—H15A	0.9600
C3—C16	1.507 (2)	C15—H15B	0.9600
C4—C5	1.393 (2)	C15—H15C	0.9600
C4—H4A	0.9300	C16—H16A	0.9600
C5—C6	1.401 (2)	C16—H16B	0.9600
C5—C17	1.500 (2)	C16—H16C	0.9600
C6—C7	1.4933 (19)	C17—H17A	0.9600
C8—C9	1.4675 (19)	C17—H17B	0.9600
C8—H8A	0.9300	C17—H17C	0.9600
C9—C10	1.392 (2)		
			
C7—N1—N2	120.73 (11)	C11—C10—H10A	119.4
C7—N1—H1A	120.4 (10)	C9—C10—H10A	119.4
N2—N1—H1A	118.9 (10)	C12—C11—C10	119.26 (16)
C8—N2—N1	114.66 (11)	C12—C11—H11A	120.4
C2—C1—C6	121.07 (15)	C10—C11—H11A	120.4
C2—C1—H1B	119.5	C11—C12—C13	120.04 (15)
C6—C1—H1B	119.5	C11—C12—H12A	120.0
C1—C2—C3	120.80 (16)	C13—C12—H12A	120.0
C1—C2—H2B	119.6	C12—C13—C14	122.03 (16)
C3—C2—H2B	119.6	C12—C13—H13A	119.0
C2—C3—C4	117.73 (14)	C14—C13—H13A	119.0
C2—C3—C16	121.07 (18)	C13—C14—C9	117.84 (15)
C4—C3—C16	121.17 (18)	C13—C14—C15	120.58 (15)
C3—C4—C5	123.35 (14)	C9—C14—C15	121.54 (14)
C3—C4—H4A	118.3	C14—C15—H15A	109.5
C5—C4—H4A	118.3	C14—C15—H15B	109.5
C4—C5—C6	117.36 (14)	H15A—C15—H15B	109.5
C4—C5—C17	120.09 (15)	C14—C15—H15C	109.5
C6—C5—C17	122.54 (15)	H15A—C15—H15C	109.5
C1—C6—C5	119.67 (13)	H15B—C15—H15C	109.5
C1—C6—C7	119.13 (13)	C3—C16—H16A	109.5
C5—C6—C7	121.19 (13)	C3—C16—H16B	109.5
O1—C7—N1	122.42 (14)	H16A—C16—H16B	109.5
O1—C7—C6	123.16 (13)	C3—C16—H16C	109.5
N1—C7—C6	114.42 (11)	H16A—C16—H16C	109.5
N2—C8—C9	120.62 (12)	H16B—C16—H16C	109.5
N2—C8—H8A	119.7	C5—C17—H17A	109.5
C9—C8—H8A	119.7	C5—C17—H17B	109.5
C10—C9—C14	119.67 (14)	H17A—C17—H17B	109.5
C10—C9—C8	120.32 (13)	C5—C17—H17C	109.5
C14—C9—C8	120.01 (13)	H17A—C17—H17C	109.5
C11—C10—C9	121.14 (15)	H17B—C17—H17C	109.5
			
C7—N1—N2—C8	170.77 (14)	C5—C6—C7—O1	−49.4 (2)
C6—C1—C2—C3	−0.8 (2)	C1—C6—C7—N1	−49.99 (18)
C1—C2—C3—C4	1.3 (2)	C5—C6—C7—N1	130.66 (14)
C1—C2—C3—C16	−176.88 (17)	N1—N2—C8—C9	176.93 (12)
C2—C3—C4—C5	−1.2 (2)	N2—C8—C9—C10	−30.9 (2)
C16—C3—C4—C5	177.01 (16)	N2—C8—C9—C14	149.45 (15)
C3—C4—C5—C6	0.5 (2)	C14—C9—C10—C11	−0.6 (2)
C3—C4—C5—C17	−178.19 (16)	C8—C9—C10—C11	179.71 (15)
C2—C1—C6—C5	0.0 (2)	C9—C10—C11—C12	1.2 (3)
C2—C1—C6—C7	−179.36 (14)	C10—C11—C12—C13	−0.9 (3)
C4—C5—C6—C1	0.16 (19)	C11—C12—C13—C14	0.0 (3)
C17—C5—C6—C1	178.77 (14)	C12—C13—C14—C9	0.6 (2)
C4—C5—C6—C7	179.50 (12)	C12—C13—C14—C15	−177.14 (17)
C17—C5—C6—C7	−1.9 (2)	C10—C9—C14—C13	−0.2 (2)
N2—N1—C7—O1	−2.7 (3)	C8—C9—C14—C13	179.43 (14)
N2—N1—C7—C6	177.21 (12)	C10—C9—C14—C15	177.45 (15)
C1—C6—C7—O1	129.9 (2)	C8—C9—C14—C15	−2.9 (2)

### Hydrogen-bond geometry (Å, º)

**Table:**

*D*—H···*A*	*D*—H	H···*A*	*D*···*A*	*D*—H···*A*
N1—H1*A*···O1^i^	0.833 (15)	2.000 (15)	2.8150 (14)	166.1 (14)
C8—H8*A*···O1^i^	0.93	2.52	3.2696 (19)	138

Symmetry code: (i) *x*, *y*−1, *z*.
